# ‘Purpose in Life’ as a psychosocial resource in healthy aging: an examination of cortisol baseline levels and response to the Trier Social Stress Test

**DOI:** 10.1038/npjamd.2015.6

**Published:** 2015-09-28

**Authors:** Nia Fogelman, Turhan Canli

**Affiliations:** 1 Department of Psychology, Integrative Neuroscience, Stony Brook University, Stony Brook, NY, USA; 2 Department of Radiology, Stony Brook University, Stony Brook, NY, USA; 3 Program in Neuroscience, Stony Brook University, Stony Brook, NY, USA; 4 Program in Genetics, Stony Brook University, Stony Brook, NY, USA

## Abstract

‘Purpose in Life’ (*Purpose*) is associated with healthy aging, but it is unknown whether this association is causal. Conceptualizing *Purpose* as a form of psychosocial resource, one mechanism promoting health could be the regulation of stress hormones. To test this hypothesis, we recruited 44 older community-dwelling adults to examine the association between *Purpose* and cortisol at baseline, in response to, and recovery from, an acute social laboratory stressor. *Purpose* did not predict cortisol baseline or reactivity, but did predict a faster recovery to pre-stress baseline levels. The health benefits of *Purpose* in aging may therefore reflect the combination of a normal stress response, which serves an adaptive benefit of allostasis, with an accelerated stress recovery, which reduces the burden of allostatic load. This model should be tested in future studies using larger samples, multiple related constructs, and longitudinal designs that include participants’ health records.

‘Purpose in Life’ (*Purpose*), a sense of meaning and goal directedness, may serve as a psychosocial resource to promote healthy aging. A high sense of *Purpose* is associated with reduced mortality,^1^ Alzheimer’s disease^2^ and myocardial infarction.^3^

One mechanism by which *Purpose* may promote healthy aging is by regulation of stress hormones, balancing the adaptive benefit of allostasis against the cost of allostatic load.^4^ We addressed this possibility using the Trier Social Stress Test (TSST)^5^ in older community-dwelling adults. We predicted that *Purpose* predicts cortisol levels at baseline, in response to, or recovery following TSST exposure.

Forty-four participants were recruited by flyers and advertisements from the area surrounding Stony Brook University. The sample was 55–90 years (*M*=65.4, s.d.=8.3; 15 males), and included Caucasian (90.9%), Hispanic (4.5%), Asian (2.3%) and African–American (2.3%) individuals. Participants were informed that the study was on stress and aging, and included saliva samples, a blood draw, psychological questionnaires and an interview-like task. Self-reported exclusion criteria were: psychiatric illness, diabetes, psychoactive drug medication, substance or alcohol abuse, smoking, hormonal medication and current immense stress. The Committee on Research Involving Human Subjects of Stony Brook University, Stony Brook, USA, approved this study.

The TSST followed a well-established protocol,^5^ involving a public speaking and math task in front of two unresponsive judges. Saliva was collected using Salivettes (Sarstedt, Germany) at arrival, 2 min prior to the TSST, and at 2, 10, 20, 30, 45, 60, 90 and 105 min post stress task. Salivary cortisol concentrations were measured in a contract laboratory (Rohleder, Brandeis University). *Purpose* was assessed using a 10-item questionnaire^1^ to measure meaning and goal-oriented feelings about the future. A 12-item version of the Trier Inventory of Chronic Stress (TICS) assessed how often participants subjectively experienced facets of chronic stress over the past 3 months.

Data analyses were conducted using SPSS v. 22 (Armonk, NY, USA). Baseline cortisol levels were log transformed to correct positive skew. Reactivity was measured as (1) area under the curve with respect to increase^6^ and (2) as peak—baseline.^7^ Cortisol recovery was the percentage difference between values at peak and 45 min post stressor: ((cortisol 45 min post stressor—peak cortisol)/peak cortisol×100). Any data point more than 2 s.d. from the mean was winsorized.^8^ Multiple regression analyses tested the association between *Purpose* and each cortisol measure, controlling for age, gender and time of day (‘morning’: 0900–1100 h; ‘afternoon’: 1100–1500 h; or ‘evening’: 1500–1730 h).

The regression model for cortisol baseline was significant (*P*<0.001), but this was driven by participants’ arrival time. The peak reactivity models were not significant. The model for cortisol recovery was significant (*P*=0.02; *R*^2^=0.288), and this was driven by *Purpose* (*β*=−0.386, *P*=0.008). Corresponding scatterplots are shown in Figure 1, and Table 1 lists all regression models.

We conducted a bivariate correlation between *Purpose* and TICS scores (representing allostatic load). This revealed that the two measures were significantly inversely related (*r*=−0.46, *P*=0.002). A regression model in which TICS scores were substituted for *Purpose* scores was not significant (*P*=0.085).

*Purpose* predicted faster cortisol recovery after a social stressor, consistent with a study reporting faster stress recovery after exposure to negative images.^9^ We found *Purpose* did not predict cortisol baseline levels or TSST reactivity. A TSST study^7^ that included *Purpose* as one element of a composite variable also found an association with cortisol reactivity but not with baseline cortisol or recovery. These findings contrast ours, perhaps due to differences in the age of study cohorts, measurement of cortisol recovery, or the use of a composite score combining *Purpose* with other constructs.

Our study has three limitations as follows: limited sample size to address related constructs, lack of objective health status, and cross-sectional rather than longitudinal design. In future research, we plan to examine overlapping constructs such as well-being, which conceptualizes *Purpose* as a subscale.^10^ In addition, although we did obtain subjective information leading us to believe that our sample was physically and mentally healthy, medical records may provide a more objective assessment. Finally, a longitudinal study would allow us to measure how *Purpose* may affect cortisol recovery throughout the aging process.

*Purpose* may facilitate stress reappraisal as we found that it was inversely correlated with the subjective experience of chronic stress (proxy for allostatic load). High subjective chronic stress on its own did not predict cortisol recovery. Therefore, our data suggest that individuals with high *Purpose* show adaptive stress reactivity (allostasis), but reduce chronic stress via cortisol recovery (allostatic load). This response style, accumulating over a lifetime of daily social stressors could be a powerful mechanism promoting healthy aging.

## Figures and Tables

**Figure 1:**
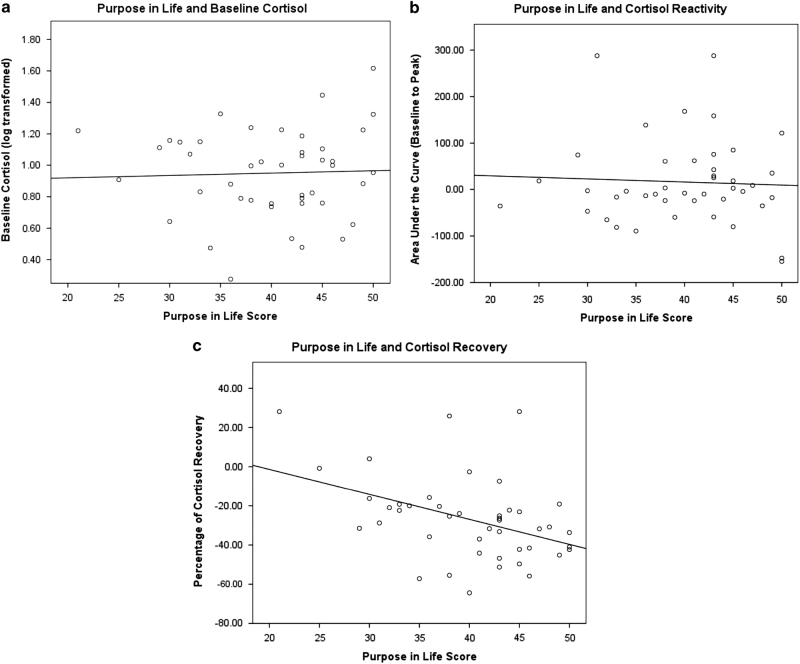
Cortisol baseline, peak and recovery as a function of ‘Purpose in Life’. The graphs above depict the relationship between Purpose in Life and baseline cortisol (**a**), cortisol reactivity (**b**) and cortisol recovery (**c**). Purpose in Life only significantly related to cortisol recovery.

**Table 1:** Model values for baseline, reactivity and recovery

	*Baseline*		*Reactivity (AUCi)*		*Reactivity (peak—baseline)*		*Recovery*	
	β	P*-value*	β	P*-value*	β	P*-value*	β	P-*value*
Time 1	−0.351	0.344	−0.125	0.465	−0.071	0.689	−0.201	0.219
Time 2	−0.657	<0.001	0.242	0.167	0.258	0.158	−0.218	0.190
Age	−0.190	0.139	−0.330	0.036	−0.258	0.108	0.156	0.282
Gender	0.088	0.488	0.044	0.773	0.081	0.607	0.221	0.131
*Purpose*	0.142	0.252	−0.083	0.574	−0.015	0.920	−0.386	0.008
Model	—	<0.001	—	0.089	—	0.283	—	0.02

Abbreviations: AUCi, area under the curve with respect to increase; *Purpose*, Purpose in Life.

Time 1 represents the afternoon group compared with the morning group. Time 2 represents the evening group compared with the morning group.
